# Effects of aqueous extract of *Hyssopus officinalis* on seizures induced by pentylenetetrazole and hippocampus mRNA level of iNOS in rats

**Published:** 2020

**Authors:** Masoumeh Gholami, Faranak Jafari, Zahra Baradaran, Jamal Amri, Hassan Azhdari-Zarmehri, Mehdi Sadegh

**Affiliations:** 1 *Neuroscience Research Center, Torbat Heydariyeh University of Medical Sciences, Torbat Heydariyeh, Iran.*; 2 *Department of Physiology, School of Paramedical Sciences, Torbat Heydariyeh University of Medical Sciences, Torbat Heydariyeh, Iran.*; 3 *Student Research Committee, Torbat Heydariyeh University of Medical Sciences, Torbat Heydariyeh, Iran.*; 4 *Traditional and Complementary Medicine Research Center, Arak University of Medical Sciences, Arak, Iran.*; 5 *Department of Physiology, Faculty of Medicine, Arak University of Medical Sciences, Arak, Iran. *

**Keywords:** Anticonvulsive, Epilepsy, Aqueous extract, Nitric oxide, Hyssopus officinalis

## Abstract

**Objective:**

We examined the effectiveness of *Hyssopus officinalis* (hyssop) aqueous extract on pentylenetetrazole (PTZ)-induced acute seizures and the hippocampus *iNOS* (inducible nitric oxide synthases) gene expression as a potential mediator of the effects.

**Materials and Methods:**

Adult male Wistar rats were used. Tonic-clonic seizures were induced by intraperitoneal (i.p.) injection of PTZ (80 mg/kg) then behavioral profile during 30 min was characterized by stages defined as seizure scores. Hyssop extract were prepared and injected (i.p.) 15 minutes before the seizure induction at three doses 50, 100 and 200 mg/kg. Experimental groups were as below: (1) saline+PTZ (n=5); (2) Hyssop 50mg/kg+PTZ (n=10); (3) Hyssop 100mg/kg+PTZ (n=10); (4) Hyssop 200 mg/kg+PTZ (n=8). Two hours after the experimental procedure, all animals were decapitated, brain was removed and right hippocampus was quickly dissected. After total RNA extraction and cDNA synthesis quantitative PCR were used for gene expression of *iNOS*.

**Results:**

Our results showed significant increase (p<0.05) in latency to reach stages 5 and 6 of tonic-clonic seizure at dose 100 mg/kg hyssop extract. In addition, this dose caused significant increase in the gene expression of *iNOS *in the hippocampus.

**Conclusion:**

It seems a 100 mg/kg dose of hyssop extract might have anticonvulsant effects. However, these anticonvulsant effects might not occur through the *iNOS* gene expression.

## Introduction

Epilepsy is a chronic, progressive and periodic neurological disorder that occurs due to sudden electrical discharge (Vezzani et al., 2011 ▶). The disease affects 70 million people in the worldwide, nearly 80% of them living in developing countries (Auditeau et al., 2018 ▶; Ngugi et al., 2010 ▶). Epileptic seizures are the result of an imbalance in the degree of excitability of cerebral areas, which occurs as abrupt and spontaneous evacuation. Brain tissue damage from trauma, and infection, and onset of inflammatory processes can be the cause of this imbalance. Temporal lobe and especially the hippocampus, are more susceptible to damage caused by epilepsy. Temporal lobe epilepsy is the most common type of epilepsy (Ahmadi et al., 2013 ▶). Finding new antiepileptic drugs is the main issue of concern because current medications have high costs, show drug resistance and produce high complications such as loss of consciousness, drowsiness, decreased white blood cell count, liver toxicity, and teratogenic effects (Theodore and Fisher, 2004 ▶; Wlodarczyk et al., 2012 ▶; Tolou-Ghamari et al., 2013 ▶). Therefore, using medicinal plants with fewer complications, which are cost-effective and available could be an appropriate alternative (Auditeau et al., 2018 ▶, Sucher and Carles, 2015 ▶). 

*Hyssopus officinalis* (hyssop) is a species of the genus Hyssopus from the family Lamiaceae. The extract of hyssop include , pinocamphone, isopinocamphone, β-pinene, and 1,8-cineole (Ma et al., 2014 ▶; Miyazaki et al., 2003 ▶). Hyssop has been used in traditional medicine to treat respiratory tract, urinary tract and gastrointestinal infections (Javadi et al., 2017 ▶). There are reports on antioxidant and anti-inflammatory effects of this plant. It was shown that hyssop reduces the expression of certain inflammatory cytokines, such as IL-1 and 17, as well as TNF-α (Özer et al., 2005 ▶). It was reported that hyssop extract is a monoamine oxidase-B inhibitor and effective in depressive behaviors (Frazer et al., 2005 ▶; Mazzio et al., 2013 ▶). In addition, the central nervous system has high sensitivity to oxidative stress due to high oxygen consumption (Li et al., 2013 ▶). Based on the available evidence, oxidative stress is recognized as a key mechanism in the pathogenesis of epilepsy (Shin et al., 2011 ▶). Oxidation stress increases the production of reactive oxygen species (ROS) and reactive nitrogen species (RNS). RNS such as peroxynitrite (ONOO^−^) are more important because they are produced from nitric oxide (NO), which has high emission power and acts as a biological messenger in the brain (Rutkowski et al., 2007 ▶; Banach et al., 2011 ▶). Nitric oxide (NO) is produced from L-arginine by the nitric oxide synthase enzyme (NOS). This enzyme has three isoforms, including brain type (nNOS), endothelial type (eNOS) and inducible type (iNOS) in immune cells, astrocytes, microglia, as well as neurons (Gonzalez-Hernandez et al., 2000 ▶; Murashima et al., 2000 ▶). It was shown that selective inhibitors of iNOS such as aminoguanidine, L-N6- (1-iminoethyl) lysine, and (-) - epigallocatechin gallate reduce epilepsy (Park et al., 2001 ▶; Byun et al., 2009 ▶).

The aim of this study was to investigate the effects of hyssop aqueous extract on pentylenetetrazole (PTZ)-induced acute seizures and hippocampus *iNOS* mRNA expression as a potential mediator of these effects.

## Materials and Methods

**Animals**

In this study, 32 adult male Wistar rats (170-200 g) were kept in 12-hr light/dark cycles. Food and water were provided freely. All experiments and animal works were performed considering the Guide for the Care and Use of Laboratory Animals (8th edition; National Academies Press; 2011) and endorsed by the Review Board and Ethics Committee of Arak University of Medical Sciences (IR.ARAKMU.REC.1397.372). All attempts were made to reduce the number of animals and their suffering. Experimental groups were as follows: (1) saline+PTZ (n=5); (2) Hyssop 50 mg/kg+PTZ (n=10); (3) Hyssop 100 mg/kg+PTZ (n=10); (4) Hyssop 200 mg/kg+PTZ (n=8). 

**Plant materials**

The air-dried aerial parts (flowers, leaves, and stems) of hyssop were purchased and authenticated by herbarium of Sabzevar University of Medical Sciences (Sabzevar, Iran). The dried plant material (50 g) was suspended in water (250 ml) and hydro distilled for 2 hr to obtain a yellowish oil. The aqueous layer from the distillate was extracted and transferred to a rotary evaporator apparatus for further extraction. Final volume of extract was almost 125 ml which means that each ml contained 400 mg of dried materials. Hyssop extract was diluted and freshly made on the day of testing using distilled water. The extract (50, 100 and 200 mg/kg) was injected (0.5 ml/rat; i.p.) 15 min (Zareie et al., 2018 ▶; Sadegh and Sakhaie, 2018 ▶) before the seizure induction. 

**PTZ-induced tonic-clonic seizures**

Acute tonic-clonic convulsions were provoked by intraperitoneal (i.p.) injection of PTZ (80 mg/kg). Immediately after that, rats were put into a plexiglass box (40×40×40 cm) as animals were visible through it and behavioral seizures were monitored for 30 min in a blind manner. For this purpose, in the experimental groups, all injections and seizure stages scoring were performed by two different persons. Consequential convulsive behaviors were categorized as follows (Luttjohann et al., 2009 ▶): stage 1, Sudden behavioral arrest and/or motionless staring; stage 2, Facial jerking; stage 3, Neck jerks; stage 4, Clonic seizure in a sitting position; stage 5, Convulsions including clonic and/or tonic–clonic seizures while lying on the belly; and stage 6, Convulsions including clonic and/or tonic–clonic seizures while lying on the side and/or wild jumping. Latency to show each stage and duration of each stage were recorded for future analyses. Also, rats were observed for mortality rate during their convulsion. 

**Molecular analysis**

Two hours after the monitoring of seizure stages for each animal, if survived following the stages 6 of convulsions, the animal was anesthetized using diethyl ether and decapitated. Brain was removed and kept in chilled 100 mM PBS (pH 7.4) and right hippocampus was quickly dissected. Total RNA was extracted using a Trizol RNA extraction kit (DNAbiotech) according to the manufacturer’s instructions. Samples were used immediately for the reverse transcription reaction using oligo-dT primers (GeneOn, Germany) and reverse transcriptase (Fermentas, GMBH, Germany) based on the manufacturer’s protocol. The reaction mixtures were incubated at 25°C for 10 min and then at 47°C for 60 min. The reaction was stopped by heating at 85°C for 5 min. Then, they were chilled at 4°C. cDNA was used for quantitative real-time PCR (q-PCR) by using a SYBER Green Master Mix Kit (Sigma,) on a Roche device (Roche, Germany). For q-PCR, the following conditions were applied: initial heating for 10 min at 95°C, 40 cycles of amplification, each for 60 sec at 95°C, 60 sec at the annealing temperature, and 60 sec at 72°C. The annealing temperatures were 55°C and 60°C for glyceraldehyde 3-phosphate dehydrogenase (*GAPDH*) and *iNOS*, respectively. To reduce the sample variations effects on results, GAPDH as an endogenous control was used. The relative changes were calculated by the comparative Cq method. Primer sequences used for amplification were designed using AlleleID 7 software (Table 1).

**Table 1:** Primers used in the gene expression study

**Gene**	**Primer sequence**	**Product length**	**Accession number**
***GAPDH***	F: TCCCATTCTTCCACCTTTGATGCT R: ACCCTGTTGCTGTAGCCATATTCAT	101	NM-017008.4
***iNOS***	F: ACCCAAGGTCTACGTTCAAGACA R: CACATCCCGAGCCATGC	121	NM-012611.3

**Statistical analysis**

Data are represented as mean±SEM. GraphPad Prism 5.0. software was used to analyze the data. Normal distribution of data was tested by D'Agostino-Pearson normality test and then One-way ANOVA followed by Bonferroni post-test was applied to evaluate statistically significant differences among more than two groups. Significance level was set at p<0.05.

## Results

Incidence of stages 5-6 and number of death in each experimental group following the stages 5 and 6, are presented in Table 2 as descriptive data.

**The effect of hyssop extract on the latency and duration of seizure stages**

**Figure 1:**
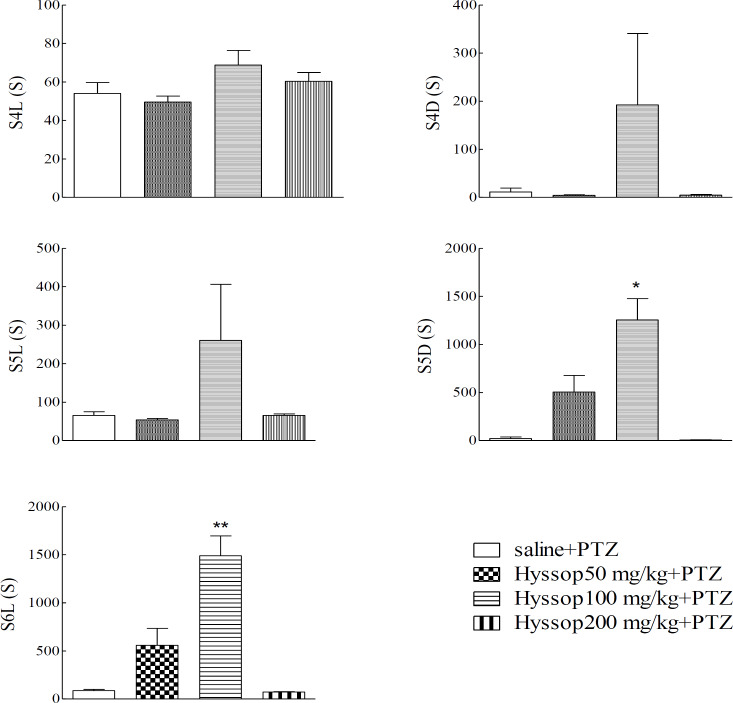
Comparison of latency (S4L, S5L and S6L) and duration (S4D and S5D) of seizure stages in experimental groups. One-way ANOVA followed by Bonferroni post-test verified that 100 mg/kg of hyssop extract has significant effects on increasing the duration of stage 5 (S5D) and subsequently increasing the latency to enter seizure stages 6 (S6L), when compared with saline+PTZ group (p<0.05). All data are presented as mean±SEM; * p<0.05 and **p<0.01 in comparison with saline+PTZ. S4L: stage 4 latency, S5L: stage 5 latency, S6L: stage 6 latency, S4D: stage 4 duration and S5D: stage 5 duration

One-way ANOVA followed by Bonferroni post-test was used to compare mean values of latency to enter seizure stages 4-6 and duration of stages 4 and 5 among the experimental groups. As shown in Figure 1, different doses of the hyssop extract (50, 100 and 200 mg/kg) did not have a significant effect on the latency to enter seizure stages 4 and 5, as well as duration of stage 4 compared to the PTZ group. 

 But, hyssop extract at the dose of 100 mg/kg was significantly effective in increasing the duration of stage 5 (p<0.05) and subsequently increasing the latency to enter seizure stages 6 when compared with saline+PTZ group (p<0.01). Also, descriptive data for mortality rates (Table 2) revealed better results following administration of the 100 mg/kg dose compared to other doses. Moreover, at the dose of 200 mg/kg of hyssop death rates were 100% during the stages 5-6. 

**Table 2:** Prevalence of stages 5-6 and number of death in experimental groups

Experimental groups	Number of rats/group	Incidence of stages 5-6	Number of death
Saline+PTZ	5	5	2
Hyssop 50 mg/kg+PTZ	10	10	5
Hyssop 100 mg/kg+PTZ	10	10	2
Hyssop 200 mg/kg+PTZ	8	8	8

**The effect of hyssop extract on hippocampus mRNA level of iNOS**

**Figure 2:**
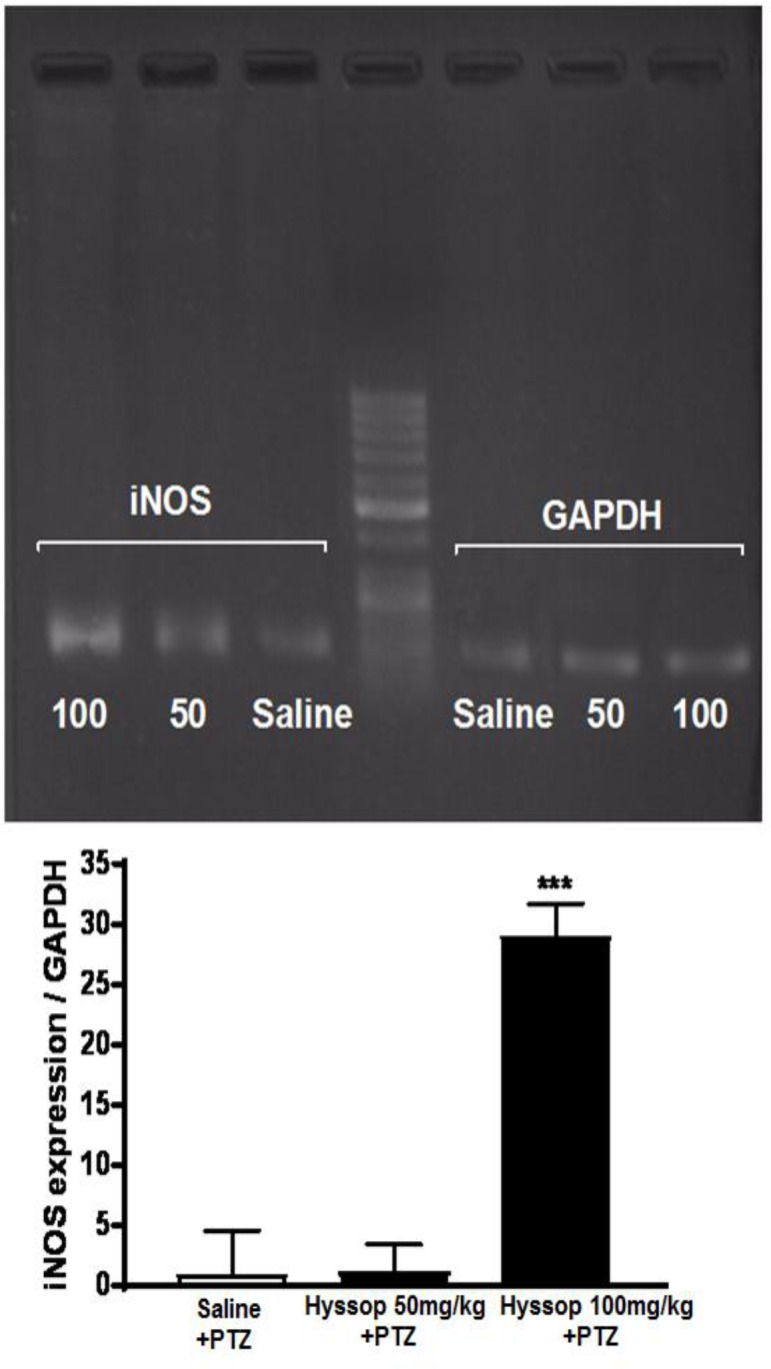
Comparison of the mean values of hippocampus *iNOS* mRNA level. A sample agarose gel (1.5%) demonstrated to compare the PCR reaction products for *iNOS* and *GAPDH* in hippocampus tissues of experimental groups (A). The molecular weights of *iNOS* and *GAPDH* are 250 and 104 bps, respectively. Hippocampus *iNOS* mRNA levels were significantly increased in rats that received 100 mg/kg of hyssop extract before the PTZ injection when compared with saline+PTZ group. However, 50 mg/kg of hyssop extract had no significant effect (B). Since, mortality was 100% in 200 mg/kg of hyssop during the seizure stages, gene expression data for this group was not achievable

Hippocampus mRNA level of *iNOS* was assessed in experimental groups after stage 6 of seizure. After performing the PCR reaction, the amplified fragment products were analyzed by agarose gel 1.5% to determine the reliability and quality of the *iNOS *mRNA status (Figure 2A). In addition, RT-PCR was performed to quantitatively measure the mRNA level of *iNOS* in the hippocampus (Figure 2B). One-way ANOVA followed by Bonferroni’s post-test was used to compare the data. As demonstrated in Figure 2B, treatment of epileptic rats with 100 mg/kg of hyssop extract led to a significant increase in the hippocampus *iNOS* mRNA level compared to the saline+PTZ group (p<0.001). However, 50 mg/kg of hyssop extract did not have a significant effect on the hippocampus *iNOS* mRNA level when compared with the saline+PTZ group. In addition, as all rats in the group treated with 200 mg/kg of hyssop, died before ending the seizure stage 6, gene expression data for this group was not attainable. 

One-way ANOVA followed by Bonferroni’s post-test was used. All data are presented as the mean±SEM. *** p<0.001 in comparison with saline+PTZ.

## Discussion

The effects of different doses of hyssop aqueous extract were evaluated on PTZ-induced acute seizures and hippocampus *iNOS* gene expression. Our results showed 

that 100 mg/kg of the extract was able to postpone the occurrence of seizure stages 5 and 6, which are concomitant with tonic-clonic attacks. Also, significant increases in the *iNOS* gene expression were observed at this dose.

Induction of acute seizures induced by PTZ injection is a common method for investigating the effects of antiepileptic drugs and chemical compounds (Löscher, 2011 ▶). PTZ works as an antagonist and inhibits the inhibitory receptors GABA_A_ in the central nervous system, which results in increased excitability of the CNS neurons. Therefore, compounds which are able to reduce this increased excitability or compounds with inhibitory effects may serve as anticonvulsive and anti-epileptic agents in this model (Schmidt and Schachter, 2014 ▶). Previous studies showed that hyssop extracts contain a high percentage of a chemical compound called iso-pinocamphone (cis- and trans-), which was extracted from aqueous or alcoholic extraction method (Millet et al., 1981 ▶). Hold and colleagues (2002) ▶ found that the pinocamphone works as an antagonist to the GABA_A_ receptor (Hold et al., 2002 ▶), so it is able to increase the brain excitability and seizure susceptibility. Regarding to those who had reported pinocamphone as a compound in both aqueous and alcoholic extracts of hyssop, the aqueous extract of hyssop in our study might be containing pinocamphone. However, its anti-seizure effects might be related to the pharmacological competition between PTZ and pinocamphone to bind GABA_A_ receptor as antagonist. In our study, the extract was injected 15 min before the PTZ and both pinocamphone and PTZ work to antagonized GABA_A_ receptors, therefore, pinocamphone may be attached to the GABA_A_ receptor prior to the arrival of PTZ and occupy PTZ binding sites or changed the affinity of the receptor for PTZ. As a result, the PTZ effects in the group receiving the extract would be lower than saline+PTZ group and delayed the occurrence of stages 5 and 6. In addition, it should be noticed that reported convulsive effects for pinocamphone in the previous studies were not in a PTZ model or seizure inducing models (Millet et al., 1979 ▶). So, it is possible that its competition with PTZ shows a different effect.

Millet and his colleagues (Millet et al., 1979 ▶) showed that non-toxic dose of hyssop extract is approximately 80 mg/kg and doses above 130 mg/kg of the extract have convulsive and toxic effects. However, we found that 100 mg/kg of aqueous extract of hyssop did not affect seizure stages 1-4. But, it increased the delay time to reach the stages 5 and 6, which means increasing the delay of tonic-clonic attacks. Therefore, it seems that 100 mg/kg of hyssop extract was able to reduce the spread of tonic-clonic seizures. However, at the dose of 200 mg/kg, these anticonvulsive effects disappeared and data from latency and duration of seizure stages changed and were more similar to data of saline+PTZ group. It is possible that the extract contains little amount of other chemicals which were not effective at low doses of extract, but when the doses grew up the effect of these compounds improved (Özer et al., 2005 ▶) and they may interact with the anticonvulsive effect of the extract. So, based on our results, it seems that the effective anticonvulsive dose of hyssop aqueous extract is 100 mg/kg and higher doses might show different effects even toxic and convulsive properties, as also previously reported by Millet et al (Millet et al., 1979 ▶; Millet et al., 1981 ▶).

Hyssop extract contains considerable amounts of linalool (Özer et al., 2005 ▶). Studies showed that anticonvulsive effects of linalool were produced by modifying the glutamate neurotransmission and reducing the system excitability (Elisabetsky et al., 1999 ▶; de Almeida et al., 2009 ▶). Therefore, at least in part, the response observed in our study might be related to the linalool content of the hyssop extract. The hyssop extract also contains flavonoid compounds (Ma et al., 2014 ▶; Özer et al., 2005 ▶). It was shown that flavonoids can act as a ligand for the GABA_A_ receptor and reduce excitation of the neurons (de Almeida et al., 2009 ▶; Wasowski and Marder, 2012 ▶). Therefore, another explanation for the results of our study could be the presence of flavonoids in the hyssop extract. 

The results of the hippocampus *iNOS* mRNA level in our study showed that 100 mg/kg dose of hyssop extract significantly increase the expression of *iNOS* mRNA. Gene expression data for the dose of 200 mg/kg are not given as all rats in this group died during the stages 5-6. However, iNOS is known as a pro-inflammatory factor which induces during the seizures and epilepsy (Murashima et al., 2000 ▶). Studies showed that suppression of *iNOS* has inhibitory effects on seizure development (Banach et al., 2011 ▶). Also, various studies showed that flavonoids and linalool and the plant extracts containing these two compounds, have inhibitory effects on iNOS, and exhibit anti-inflammatory effects by reducing NO production (Peana et al., 2006 ▶). Therefore, our results showed contradictory findings with previous reports and despite the extract’s anticonvulsive effects, hippocampus *iNOS* gene expression increased. These unexpected contradictory effects might be produced by different chemicals present in the extract. Furthermore, it should be notified that the observed anticonvulsive effects of hyssop extract and its probable interaction with GABA_A_ which was discussed earlier, occurred fast as we injected PTZ 15 min after the injection of hyssop extract. But, *iNOS* gene expression data was collected approximately 3 hr after the hyssop extract injection. As we know inducing the gene expression needs time and conversion of this gene expression to the physiological results also needs time. Thus, as these two contradictory data, anticonvulsive effect and increasing the *iNOS* gene expression, might not interact as they did not occur at the same time. However, it needs to be investigated, if the anticonvulsive effect of hyssop is time-dependent, and if there is a possible convulsive effect in a prolonged timeline or in chronic administration due to changes in *iNOS* gene expression. 

It seems that 100 mg/kg of hyssop extract has anticonvulsive effects. However, these anticonvulsant effects are not mediated through the inhibition of *iNOS* gene expression.
